# Leadership, institution building and pay-back of health systems research in Mexico

**DOI:** 10.1186/1478-4505-7-22

**Published:** 2009-09-30

**Authors:** Miguel Angel González-Block

**Affiliations:** 1 Health Systems Research Center, National Institute of Public Health, Cuernavaca, Morelos, Mexico

## Abstract

**Background:**

Health systems research is being increasingly called upon to support scaling up of disease control interventions and to support rapid health sector change. Yet research capacity building and pay-back take years or even decades to be demonstrated, while leadership and institution building are critical for their success. The case of Mexico can be illustrative for middle income countries and emerging economies striving to build health research systems.

**Methods:**

Historical reflection suggests the relationship between health sector reforms and economic crisis, on the one hand, and research capacity building and payback, on the other. Mexico's post-revolutionary background and its three health sector reforms are analyzed to identify the emphases given to health systems research.

**Results:**

The first wave of health reform in the 1940s emphasized clinical and epidemiological research. Health systems research was not encouraged in a context of rapid economic development and an authoritarian regime. In contrast, health systems research was given a privileged place with the second wave of health reforms in the 1980s, which addressed health system coordination, decentralization and the universal right to health in a context of a deep economic crisis. The third wave of health reforms between 2003 and 2006 was based on the health system models proposed through research in the 90s. The credibility gained by research institutions was critical to ensure government uptake. Research influence can be traced through the role it played in defining a problem, in designing innovative insurance mechanisms and in establishing evaluation frameworks. It is argued that the Ministry of Health's budget increase of 56% between 2003 and 2006 and the reductions in inequity are pay-back to research investments since the 1980s.

## Introduction

Health systems research requires a special kind of leadership and institution building to fulfill its mission of producing knowledge and applications to improve the way in which society organizes itself to attain health objectives. Leadership is required to develop the institutions and culture that lead to valuing critical knowledge and innovation in the process of attaining health objectives. Demand for research and critical analysis are thus as essential to the definition of health systems research as is its supply through appropriate methods [1]. Strengthening health systems research leadership thus requires understanding the context in which research can thrive.

Health systems research is particularly valuable during the process of formulating and implementing health sector reforms. Comprehensive health reforms imply important and rapid changes in how societies organize their response to health problems, including changes at the systemic, programmatic, organizational and instrumental levels [2]. Health reforms can be characterized in terms of new contents, sequence in a historical context, implementation processes, policy purpose and scope. At its most general, reform refers to the removal of evil or corrupt elements out of the body politic, or to the willed evolution of the social system towards "better" stages of being [3]. Three generations of reforms have been widely identified, starting with bureaucratic reforms specially after the Second World War, followed by regionalization and decentralization reforms emphasizing primary health care in the 70s and 80s, and followed by what have been termed "third generation" reforms where demand-based financing and incentives are being introduced to respond to increasingly complex health needs and the political imperative of universal health service coverage [4].

Based on interviews with key players, participant observation and the review of research and policy publications, this paper analyses the development of health systems research in Mexico from a long-term perspective and at the macro-institutional level. In a broad sweep, the research institutions and expectations are outlined, particularly between the first and third waves of health reform spanning the 1940's to the 2000's. The long-term perspective enables a unique understanding of research institution building in its relationship to health reforms. This perspective also reveals the payback that research on health systems and on population health can give in the medium to long term. The macro-institutional perspective allows an understanding of the importance of building relationships of solidarity across agencies as well as between the national and international levels that are so important for the attainment of the mission of public health, and particularly for global health. It is in this light that we can better understand the intricate relationship between personal leadership, experience and vision and the opportunities that specific economic and social situations afford in the creation of health research institutions.

### Public health capacity building and the role of international agencies

The School of Public Health of Mexico (SPHM), was founded in 1922 out of the government's interest to make modern, scientific public health the spearhead of development. However, program development was informed mostly through research and trends imported from the United States. This process was supported by the Rockefeller Foundation's fellowship program, which between the 1920s and 50s produced 67 Masters and one Doctor of Public Health, mostly at Johns Hopkins University. Fellows were motivated by the Progressive Era's confidence in the ability of science to systematically solve society's problems [5]. However, most of these fellows were focused on applying existing knowledge rather than engaging in new research. In this context, the blueprint for a federal public health model was closely based on the experience of municipal public health in the United States. Medical care was not included at this time as a government priority, leading to the emergence of a disorderly, corporatist scheme of subsidized medical care in support of key industry workers.

With World War II Mexico experienced rapid industrialization, seizing on the opportunity for import substitution and market protection. In this context, the government embarked in the country's first wave of health reforms to consolidate and balance the federal and the corporatist approaches and to coordinate public health with medical care. Two complementary institutions were created in 1943: the Ministry of Sanitation and Assistance (SSA) to strengthen public health and medical care for the poor under a federal model, and the Mexican Institute of Social Security (IMSS), consolidating the corporatist model to provide the best medical care available to formal sector workers spearheading development. At the same time, private medical care was encouraged as a means to fulfill the demand for specialized medicine as well as to provide an outlet for unmet needs. The State thus institutionalized the health policy principles of charity, privilege and the market, relegating citizenship and therefore equity.

Clinical research was first instituted in this context through an enlightened charity principle that proposed to address the immediate effects of poverty through medical care as well as the understanding of its biological determinants through research. Import substitution under a state controlled economy promised fiscal support for a massive hospital building program, while the first, research oriented health institutions were created: the Children's Hospital and the National Institute of Nutrition.

The institutional bases of public health training were also transformed in this context. RF's fellowship program was sharply reduced in Latin America and SPHM took over the program by recruiting full time staff and adopting RF's curriculum, fellowship model and prestige. The government and the Pan American Health Organization responded with scholarships, turning the old institution into a nationally and internationally recognized School of Public Health. In a context of growing government centralization and investments in disease control, SPHM was consolidated as a field-based, pragmatic, interdisciplinary and federally oriented public health training enclave, quite different to -- and isolated from -- academic postgraduate institutions and clinically oriented research hospitals. SPHM was thus fully dedicated to producing the growing number of public health professionals that Mexico and other Latin American countries required, with little attention to research.

However, the country was witnessing increasing problems in coordinating vertical disease control programs and the also in the growing overlap in primary health care services provided by SSA and IMSS. Growing concerns fueled the establishment of the journal Salud Pública de México in 1959, giving program officers and policy makers the opportunity to address health needs and to inform on general aspects of program performance.

### Research capacity building in the second wave of health reforms

Growing overlaps in medical infrastructure and responsibilities across institutions in a context of economic stagnation in the 1970s soon alerted government to costly inefficiency and to governance problems. Furthermore, the Ministry's public health model had become a tangle of vertical and special programs working at cross-purposes. To address these wide ranging problems, the federal government established in 1981 the first sector-wide Commission to restructure the health sector, under the leadership of Guillermo Soberón [6]. Soberón had just left a highly successful presidency of the National Autonomous University of Mexico (UNAM), characterized by the growth of research institutions and public-private partnerships. The Commission went on to produce a great deal of evidence and a will towards reform, but also manifested a lack of health systems research and analytical capacity within government.

Stagnation in the growth of health services given economic slowdown fueled interest in social medicine and in community health. Interest in research and critical analysis was manifested in UNAM as a strategy to innovate and transform the health sector. Backed by scholarships financed through expanded oil revenues, several young medical graduates went on to seek doctoral training in public health abroad. A postgraduate social medicine program was also started in the new Autonomous Metropolitan University, influenced by European universal health care models and trends in social epidemiology in Latin America. SPHM could not remain isolated from the winds of change and started its first program to strengthen research capacity in collaboration with an external medical anthropology research initiative.

Mexico's second generation of health reforms in the mid-eighties were influenced both by the rationalization efforts within the government sector and by the critical winds blowing within the academic sector. Given his immediate experience in both sectors, Guillermo Soberón was appointed Health Minister in the 1982 federal administration. He proceeded to promote a Constitutional amendment recognizing the Right to Health, thus legitimizing the Ministry's role as steward of the health sector. The Right to Health privileged the citizenship principle and thus subsumed the prior corporatist right to health. However, the new arrangement left intact social security's laws and functions, complete with their own regulation, financing and service provision. Furthermore, just as the new government pushed these reforms, Mexico witnessed the start of a deep economic recession. Between 1982 and 1985 public health sector spending was reduced by 25.5% and real wages declined by 30% [7]. The Ministry of Health's main challenge was thus focused on internal reform, setting about to integrate public health programs as well as medical operations at the state level through decentralization. Research was to be critical in this enterprise.

Research was trusted by Minister Soberón to provide the necessary evidence to consolidate sector-wide stewardship, secure universal access to heath care and integrate program management, all in a context of reduced health sector funding. The centerpiece of this effort was the establishment in 1984 of the Center for Public Health Research (CPHR) within the Ministry, as a 15 person-strong agency in a complementary relationship to SPHM [8]. The Minister appointed Julio Frenk as CPHR director, who was one of the doctoral graduates he had got to know as medical students at UNAM, and whose postgraduate careers he had followed. This was no easy task, as the young graduate had already gained a professorial appointment in the US and, given the deep economic crisis, was about to be lost to brain drain. The Minister was able to repatriate Frenk together with other young graduates thanks to a clear vision for research that included institution building as part of health sector reform. Importantly, the CPHR convened right from the start an International Advisory Committee bringing together the foremost public health experts and institution builders from Mexico, US, Canada, England and Latin America. This group was to prove of strategic value to help steer new projects and to relate them to international efforts. They would also play a critical role in legitimating CPHR in the face of its detractors.

The founding group of CPHR researchers launched an ambitious program focusing on fundamental topics such as the epidemiologic transition, the quality of care, the effectiveness of primary care interventions, and the determinants of physician supply and employment [9]. Given the scarcity of national funds, these were obtained from foundations and international organizations, which allowed for further recruitment of researchers and purchase of computers. Among the early achievements of CPHR was the seeding within SPHM of the first research-oriented postgraduate program in the country, the Masters in Science in Health Systems, generously funded by the Kellogg Foundation.

CPHR was able to demonstrate its value immediately after its foundation. Mexico City experienced, in September of 1985, devastating earthquakes that produced a severe loss of health care infrastructure, including 5,000 hospital beds. The Center rose to the occasion and carried out a comprehensive study of reconstruction options which led, among other outcomes, to the design and implementation of advanced primary care facilities capable of addressing hospital capacity shortages. CPHR was also tasked with evaluating the process and early impact of decentralization efforts, leading to identify its benefits in the midst of economic crisis and in a context of political debate regarding its wider institutional impacts [10]. The ability to respond creatively to economic and natural catastrophes and to the challenges of policy implementation, while at the same time maintaining the highest scientific standards, firmly established the reputation of CPHR [11,12].

One of the major challenges for CPHR was to obtain the legitimacy, recognition and support of two constituencies that had traditionally been skeptical about the value of public health research: on the one hand, decision makers, who often believe that research does not address their needs; on the other hand, the biomedical research community, which may look down on the scientific rigor of public health. To address this challenge, CPHR combined two guiding principles: **relevance **to decision making with **excellence **in the academic quality of its products [13]. Mechanisms and tools were developed to bridge the divide between researchers and policy makers, such as joint seminars, priority-setting exercises, executive summaries and a revolving door between the two communities.

After three years of operation CPHR and SPHM had learnt to interact and were able to accommodate their different missions and orientations. Minister Soberón also understood the importance of consolidating public health research and teaching within a solid, well-recognized institution. In spite the fact that the economic crisis continued unabated, the minister convinced the President of Mexico to decree the establishment of the National Institute of Public Health (INSP) in 1987, confederating the public health teaching and research ministerial agencies together with a nascent Centre of Infectious Disease Research. INSP was made the tenth within the cadre of Mexico's well recognized national institutes of health, a strategy that no doubt helped to establish the prestige of public health research within the health sector as well as attract the talent needed for the Institute's future.

#### Participation of the private sector

The economic crisis threatened right from the start of the new administration in 1983 the country's drug supply and the development of the national pharmaceutical industry. This challenge could easily have detracted from any efforts at building public health research capacity. Far from it, this complex scenario was seized upon by the Minister as one more opportunity in his design for institution building. Several of the most enlightened business leaders were persuaded to endow in 1985 the Mexican Health Foundation (Funsalud) as an evidence-based policy formulation think-tank and a stronghold to support health research capacity building. FUNSALUD went on to promote strategic alliances for policy dialogue and development with academic institutions, the private sector, government, international organizations, civil society and other foundations abroad.

FUNSALUD supported since its inception health research by managing research funding for third parties and addressing "brain drain" by funding the return of researchers. It also stimulated national researchers through health research prizes. FUNSALUD and INSP developed many collaborative initiatives, some of which would be seminal to steer Mexico's third wave of health reform and to influence health systems development internationally. This was made possible in part through the appointment of Minister Soberón as the Executive President of FUNSALUD at the end of his term, as well as through the exchange of staff members through the years [14].

The strategic value of collaboration between INSP and FUNSALUD was proven when the ambitious second wave of health reform started in 1983 came to a standstill in 1989, when the decentralization process was interrupted. Furthermore, health policy research came to be perceived as a threat by the Ministry of Health, in the context of its diminished power to steer the health sector as a whole. This challenge was seized upon as an opportunity by INSP, which went on to develop collaborations with academic and health service institutions in selected states to decentralize health systems research. INSP's Founding Director went on to become Funsalud's Executive Vice-President after his term ended, with the aim of launching the Health and the Economy Study. Supported by half a million dollars from FUNSALUD's own members, this ambitious undertaking carried out, for the first time in a developing country, a comprehensive analytical exercise including the measurement of the burden of disease, cost-effectiveness analysis of interventions national health accounts, and political mapping. While these tools had been initially developed mostly by international organizations on the basis of partial data, they were first tested and refined in Mexico as part of this effort. Funsalud's modest investment and leadership contributed international public goods that would later exert influence on a much wider scale.

Perhaps of greatest importance, the Health and the Economy Study enabled the reflection of the significance, reach and limitations of the various waves of health sector reforms in Latin America and in developing countries as a whole. In collaboration with WHO, Funsalud and INSP jointly established the International Clearinghouse of Health Sector Reform Initiatives. At the same time, Funsalud participated in the Ad Hoc Committee on Health Research Priorities Relating to Future Intervention Options. These efforts would be key for the establishment in 1999 of the Global Forum for Health Research and in 2000 of the Alliance for Health Policy and Systems Research. The Health and the Economy study was also critical in developing the model of structured pluralism to guide the functional integration of the social security and ministry of health models that characterize most Latin American health systems.

Thanks to its wide recognition, the Health and the Economy study led to the establishment of the Centre for Health and the Economy within FUNSALUD and to the publication of the "Health Observatory", a series of highly influential and innovative statistics, analyses and recommendations. This body of work and experience established a credible link between research and policy making in Mexico. This influence would be extended internationally with the appointment of Funsalud's Executive Vice-President as the first Executive Director of the Evidence and Information for Policy Cluster established by Brundtland's WHO administration in 1998. Indeed, the influence of the Health and the Economy study can be seen in the conceptual framework of the World Health Report 2000 "Health systems: improving performance" [15] and in the design of the Commission on Macroeconomics and Health chaired by Jeffrey Sachs.

#### Health systems research consolidation at INSP

In its 20 year history, INSP saw a growth of its critical mass of academic staff from 20 in 1987 to 170 in 2008. This growth enabled the consolidation of a wide range of fields, to become among the world's most diversified public health research and development institutions. The SPHM was integrated to the research centers in 1995 as a strategy to strengthen teaching within research centers, thus upgrading its overall quality. INSP concentrates the largest capacity in health systems research in the country and grew from 5 researchers twenty years ago to 43 today. At the same time, the Center for Health Systems Research was established, integrating the social science teaching and research that had existed within SPHM together with health policy analysis. This allowed the establishment of research lines on the social response to the health needs of vulnerable populations, such as migrants, indigenous peoples and the elderly. Health policy research was strengthened in areas such as human resources and pharmaceuticals, decentralization, comparative health policy analysis, social protection in health, system governance and equity.

#### Pay back from health systems research in the third wave of sector reforms

The pay back of public health research supported by the Ministry of Health of Mexico and its leaders can be traced through its influence in research targeting and capacity, in policy structures, processes and results, and in wider health and economic benefits [16]. Research targeting was benefited at both international and national levels, as Adolfo Martínez Palomo, a distinguished Mexican health researcher, participated in the Evans Commission, which was to establish the Council on Health Research for Development (COHRED) from 1991. This effort led to the establishment in Mexico of the Mexican Commission on Health Research (COMISA), which was to play a key role in the development of cross-institutional understanding on the importance of this endeavor.

Policy making structures were directly benefited by research as the leadership of INSP and FUNSALUD were recognized shortly after the historical election of 2000, which ended single-party dominance in the country. At that point, Julio Frenk was appointed Minister of Health. Not surprisingly, the Ministry's plan to take the health reform forward in Mexico drew heavily on research models and data. Research evidence was made one of the three foundations of reform, together with ethical deliberation and political negotiation. Policy research was used to set a strategy to realign and coordinate SSA and IMSS, among other public institutions, taking them from multi-functional monopolies and towards specialized agencies in charge of stewardship, financing and provision. Seguro Popular was launched as a scheme to universalize essential health services and to ensure that all Mexicans were protected from catastrophic health expenditures. The Health and the Economy study provided the bases for critical research inputs. National health accounts identified catastrophic health expenditure as a critical problem and thus as the main driver of health insurance. The structure of private contributions and private expenditures was identified as the main challenge for equity and efficiency; cost effectiveness analysis was used to develop a package of universal health interventions, and institutional and financial analysis was developed to set the architecture of Seguro Popular agencies at state level. Evaluation was strengthened within the Ministry of Health to make federal and state health authorities more accountable to Congress and to the population at large [17].

Health systems research supported the Ministry of Health in gaining trust as a steward of the health system under a democratic regime. Even the staunchest critics of Seguro Popular acknowledge the role that research played in its formulation and devote important efforts to discuss possible weaknesses in data collection, analysis and interpretation [18]. Congress, while critical also of several features of Seguro Popular, was persuaded with research evidence that this policy was a sound and fiscally responsible investment to ensure social and economic development, and to set the foundations for the functional integration of the health system. On this basis, Congress authorized a major budget increment of one percentage point of GDP between 2004 and 2010 to achieve universal coverage through Seguro Popular. Policy implementation also benefited greatly from research investments, both through the participation of researchers in the development of diverse platforms and tools, and through studies to refine policy estimates and to evaluate processes and early results. Thanks to this support, between 2003 and 2006, the Ministry's budget increased an unprecedented 58% or USD 3.3 billion overall. The per capita health expenditure differences between the social security beneficiary in the formal sector and the non-insured (self-employed or informal sector worker) has been reduced, from 2.44 in 2003 to 1.75 in 2006.

This Ministry's budgetary increase, and its equity impact, can be conceived as a good indicator of the payback from health systems research investments in the medium term. The FUNSALUD study "Health and the Economy", published in 1994, was arguably the single most important contribution to future policy, at a total cost of half a million dollars, a mere fraction of the financial impact it wrought ten years later. This investment should be placed in the context of health systems research investments between 1984 to 2003. In this period, roughly 500 researcher years were invested at CPHS, INSP and Funsalud, at a cost of about USD 350 million, about 10% of what the third wave of health reform achieved financially during its implementation years. Health systems research is clearly an excellent investment, albeit one that matures only in the long term and requires a great deal of policy stability and perseverance.

Early investments in health systems research have also paid-off in terms of the spread of the field across other institutions, although with a greater clinical focus. Thus, 22% of projects funded by Mexico's Health Sector Research Fund between 2002 and 2006 address the field, for a total investment of USD 8 million or 30% of total funding. This funding was greatly complemented during the period through expenditures by the Ministry of Health for national surveys and mandated program evaluations, exceeding USD 20 million.

### New directions

In spite of important growth, health systems research is highly concentrated in a handful of institutions. While this concentration could have been warranted during the capacity building stage, the third wave of health reforms has given state governments increased funds to allocate with a high degree of autonomy. All states therefore require research capacity, and yet most of them lack it almost completely.

INSP is addressing the concentration of research capacity through a multi-pronged strategy. Health systems research capacity is being built at INSP's facility in Chiapas, catering for the poorest states in the south. A national consortium of academic institutions was established to strengthen state level research capacity, and particularly the capacity of policy makers to utilize research. Interventions are under way to assess the capacity to utilize research, to develop priority setting tools and processes and to develop local research networks capable of taking up the challenges. Research brokers are also being trained and instituted in collaboration with non-government organizations.

Private sector funding and strategic orientation of health systems research and development is now poised for a strong comeback in Mexico through Instituto Carso de Salud. This initiative is developing a collaboration with the Institute on Health Metrics and Evaluation to strengthen this important research and analysis area for Latin America.

## Conclusion

Investments in health systems research in Mexico at least since the second wave of health reforms in the mid-eighties contributed to the reach and credibility of health policies and programs in Mexico and internationally. Decision makers have today a clear picture of the challenges and issues still facing the country, particularly in progressing towards a truly universal health system that recognizes the value of citizenship, while ensuring support to economic and social development. Decision makers are demanding research at all levels and are ready to invest increasing resources to obtain critical feedback on their policies and programs. Health systems research is now demonstrating important pay-back in access to health services and particularly in health equity.

Mexico's mid-term experience with institution building for health research and its focus on health systems research is a case study for global health programs. Research institutions have provided stability to investments as well as the capacity to transfer experiences. Mexico's policy continuity and relative stability have been important for institution building. It is also now a basis on which to build South-South collaboration with Latin America and beyond, extending now the benefits that Mexico obtained from international collaboration in the past. Mexico is also poised now for modest efforts in South-North collaboration, where issues such as the health needs of millions of international migrants can be addressed through leadership from the South.

## Competing interests

The author declares that they have no competing interests.
